# The Profile of Markers of Bone Turnover, Inflammation and Extracellular Neutrophil Traps on Bone Mass in Haemophilia and the Development of Haemophilic Arthropathy

**DOI:** 10.3390/jcm11164711

**Published:** 2022-08-12

**Authors:** Sylwia Czajkowska, Joanna Rupa-Matysek, Ewelina Wojtasińska, Kacper Nijakowski, Anna Surdacka, Lidia Gil

**Affiliations:** 1 Department of Conservative Dentistry and Endodontics, Poznan University of Medical Sciences, 60-812 Poznan, Poland; 2 Department of Haematology and Bone Marrow Transplantation, Poznan University of Medical Sciences, 60-569 Poznan, Poland

**Keywords:** osteocalcin, alkaline phosphatase, haemophilia, bone remodelling

## Abstract

Background: The aim of the study is to evaluate selected biomarkers of bone turnover, inflammation, neutrophil trap and factors predisposing haemophiliacs to bone loss, and to analyse their relationship with clinical features, treatment and complications. Methods: The levels of interleukin 6 (IL-6); citrullinated histone (CH3); osteocalcin (BGLAP); bone alkaline phosphatase (BALP); N-terminal procollagen type I propeptide (P1NP); and C-terminal collagen type I telopeptide (C1CP) were examined in 60 patients with haemophilia. Results: The cut-off value for BGLAP is 26.41 ng/mL, and 929.7 pg/mL for CH3. There is a statistically significant difference between BGLAP, BALP, C1CP and CH3 concentrations, depending on the prophylaxis used. The median concentration of BGLAP in patients taking the factor on demand is 28.0 ng/mL, BALP 322.5 U/L, C1CP 191.2 ng/mL and CH3 1114.4 pg/mL. In patients taking recombinant coagulation factor VIII/IX as prophylaxis of bleeding, the median BGLAP concentrations are 35.9 ng/mL, BALP 280.9 U/L, C1CP 161.6 ng/mL and CH3 952.5 pg/mL. BGLAP and BALP concentrations are dependent on the development of haemophilic arthropathic. Conclusions: The concentrations of selected markers of bone turnover and NETs may help to identify patients at particular risk of developing haemophilic arthropathy and bone metabolic turnover abnormalities.

## 1. Introduction

Haemophilia is one of the most common plasma haemorrhagic diatheses and is inherited in a recessive, sex-linked manner. The clinical picture of haemophilia depends mainly on excessive or spontaneous bleeding, which leads to the formation of secondary complications such as pseudotumours [1,2,3,4], or haemophilic arthropathy [5,6,7,8,9] and secondary muscular atrophy [8]. The consequence of the development of haemophilic arthropathy may be an imbalance of bone metabolism and a decrease in bone mass [10,11,12,13,14]. However, the decrease in bone density depends on many factors and so far, no mechanism has been found for the development of osteoporosis and osteopenia in haemophilia. The literature has repeatedly attempted to find a relationship between haemophilia and bone loss [15,16,17,18,19], which would make it possible to reduce the incidence of osteoporosis by eliminating risk factors, and thus improve the quality of life of patients with haemophilia. It is well known that bone mass depends, inter alia, on physical activity, so it should correlate with the efficiency of the joints, but also with the possible fear of trauma and bleeding, which may occur in patients with congenital bleeding diathesis [20]. The literature also suggests that there may be a direct relationship between factor VIII and IX and bone resorption [21,22,23], as well as between the development of osteoporosis and infection [24], which may occur due to the use of blood products [25,26]. In addition, decreased bone density may be associated with vitamin D deficiency [27] (related to the geographical location and climate of the inhabited region), decreased serum total calcium levels, or be dependent on the level of proinflammatory cytokines. However, the studies conducted so far do not answer all the questions and do not clearly indicate the most important factors predisposing to the development of osteoporosis in haemophilia. Finding a predictive marker that indicates an increased risk of arthropathy or imbalance of bone metabolism in haemophiliac patients is essential to improving the quality of life in this group.

Bearing in mind the above-mentioned complications, primarily the reduced bone mineral density and arthropathy, the authors aimed to assess the concentrations of selected biomarkers of bone turnover, the neutrophil extracellular trap marker and inflammatory markers in patients with congenital haemophilia. The aim of the study is to assess the concentrations of these biomarkers in the blood of patients with haemophilia A and B and to analyse their relationship with clinical features, treatment and complications. Changes in the concentrations of bone markers reflect the dynamics of bone turnover and have diagnostic significance in the case of unbalanced bone remodelling, which may be key to understanding the mechanism of bone loss in haemophilia. Interleukin 6 (IL-6) is the main factor regulating the body’s defence mechanisms [28]. It is involved in a number of physiological and pathological processes, including the response to trauma or infection. It influences the development and progression of inflammation, but, importantly, also affects osteoclastogenesis [29]. Neutrophil extracellular traps (NETs) also play an important and constantly studied role in fighting infection. Recently, there has been increasing postulation about the collateral effects of NET formation, including pathologies related to the coagulation system. Recent studies have reported a possible influence of extracellular neutrophil traps on the pathogenesis of haemophilic arthropathy [30]. However, the impact of NET formation on the course and development of complications in haemophilia is not known, nor has the relationship between NET markers and the type or form of haemophilia been studied. Moreover, the pathogenesis of osteoporosis and osteopenia in haemophilia remains unclear due to the possible influence of many factors on decreased bone mass. Among other things, it is not known to what extent intra-articular bleeding and the development of arthropathy reduce bone mass and, thus, whether the form of haemophilia or coagulation factor prophylaxis significantly affects bone turnover. There is also the problem of distinguishing among patients with haemophilia the group that is particularly at risk of developing osteoporosis, in order to prevent its development.

## 2. Materials and Methods

The study was conducted according to the criteria of the Declaration of Helsinki. The approval of the local Bioethics Committee (no. 628/20 with amendments no. 210/21) and the written consent of the patients to participate in the study were obtained prior to the study. The cross-sectional study included 60 patients aged 18 to 69 years under the care of the Department of Haematology and Bone Marrow Transplantation who were diagnosed with haemophilia type A or B with mild, moderate or severe type, on the basis of clinical features and currently available laboratory tests (Table 1). The median age was 36 years. All patients were treated “on demand”—a factor administered in the event of excessive bleeding/trauma (45%)—or received prophylactic clotting factor (55%). The patients have regular check-ups at the haemophilia treatment centre and periodic orthopaedic consultations to qualify for further surgical treatment and intensive rehabilitation. Haematological checks take place at least every three months depending on joint complaints, bleeding or other complications of treatment. In Poland, prophylactic treatment in adult patients is based on regular injections of plasma-derived factor concentrates, in accordance with the “National programme for the treatment of patients with haemophilia and related haemorrhagic diathesis for the years 2019–2023”. Most patients who developed haemophiliac arthropathy underwent joints alloplasty. The total mean annualized bleeding rate reported was 8 bleeds, ranging from 5 to 15 in the group treated prophylactically for 1 year, and up to 30 bleeds for the group on-demand. The control group consisted of 30 men aged 21 to 63 years.

From all patients, 10 mL of peripheral blood was collected once into vacuum tubes containing EDTA for determination by commercial ELISA (Shanghai Sunred Biological Technology Co, Shanghai, China), as well as concentrations of interleukin 6 (IL-6); citrullinated histone CH3 (a marker of neutrophil extracellular trap); markers of bone formation (osteocalcin (BGLAP), bone alkaline phosphatase (BALP), N-terminal propeptide of type I procollagen (P1NP)); and the bone resorption marker C-terminal telopeptide of type I collagen (C1CP). Venous blood samples were collected in the morning to avoid diurnal variations in concentrations. The samples were stored at −70 °C to −80 °C until assays were performed. Tests were performed according to the manufacturer’s instructions.

From the available medical records, information was obtained on the treatment of haemophilia; viral status (HIV, HBV, HCV); occurrence of haemophilic arthropathy (physical examination); and concentrations of vitamin D, ferritin, serum calcium and CRP (tests performed in the haematology clinic on the day the blood samples were secured). No patient was HIV-positive, 24 patients had a history of hepatitis B virus (HBV), and 28 patients had a past or current history of hepatitis C virus (HCV). HCV RNA was detected in the plasma of three patients.

Patient inclusion in the study was conditional on being above 18 years of age. The patients were randomly selected. A condition for exclusion from the control group was a history of any factors that might predispose a patient to decreased bone mass (including thyroid and parathyroid dysfunction, or long-term steroid intake).

The data were analysed using Statistica version 13.3 (Statsoft, Cracow, Poland). The significance level was set at α = 0.05. To compare differences between groups the Mann–Whitney U test for continuous variables was used. The test used to compare more than two variables was the Kruskal–Wallis test. ROC curves were used to find the optimal predictive model.

## 3. Results

**Figure 1:**
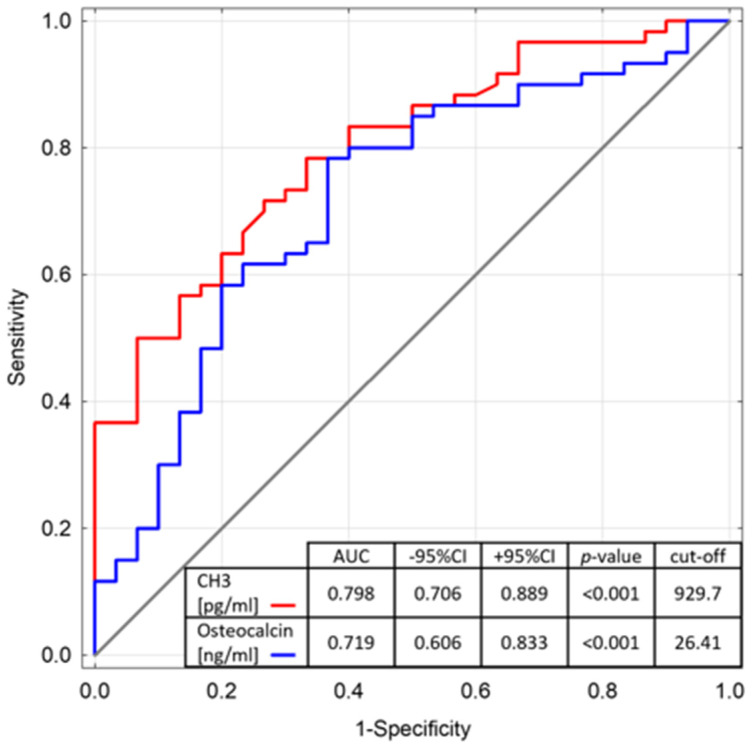
The receiver operator characteristic curve for osteocalcin (BGLAP) and citrullinated histone (CH3) levels for classification of patients with and without haemophilia (Youden’s Index). AUC—the area under the ROC curve; CI—confidence interval.

The results of the study are presented using tables and graphs. Table 2 presents the test probabilities (*p*-value) and medians, and the upper and lower quadrants for the concentrations of individual markers (interleukin 6, citrullinated histone CH3, osteocalcin, bone alkaline phosphatase, N-terminal propeptide of type I procollagen, and C-terminal telopeptide of type I collagen) in the study and control groups. Statistically significant differences were observed in the concentrations of citrullinated histone CH3 and osteocalcin. The cut-off values for concentrations of citrullinated histone CH3 and osteocalcin were obtained using ROC plots (Figure 1).

Table 3 shows the comparison of concentrations of markers of bone turnover (osteocalcin, bone alkaline phosphatase, N-terminal propeptide of type I procollagen, C-terminal telopeptide of type I collagen, calcium, vitamin D); inflammation (interleukin 6); neutrophil trap (citrullinated histone CH3); and ferritin, according to the prophylaxis used (prophylactic/“on-demand” factor). The therapy used affected the blood levels of citrullinated histone CH3 osteocalcin (BGLAP); bone alkaline phosphatase (BALP); and C-terminal telopeptide of type I collagen (C1CP).

Table 4 refers to the concentrations of the studied markers (medians, upper and lower quadrants) in patients with mild, moderate and severe haemophilia, and factor VIII/IX levels below 1% of normal (severe haemophilia) and above 1% of normal (moderate and mild haemophilia).

There were no statistically significant differences in the values of concentrations of the tested markers depending on the type of deficient clotting factor (haemophilia A or B), but there was a tendency towards a higher blood concentration of osteocalcin in patients with haemophilia B, compared to patients with haemophilia A (data not presented).

There was a correlation between the concentrations of selected markers of bone turnover (osteocalcin, acid alkaline phosphatase) and interleukin 6 and the development of haemophilic arthropathy (Table 5).

The influence of virological status (HBs antigen, HCV antigen) on selected markers of inflammation, bone turnover, ferritin and extracellular neutrophil trap is presented in Table 6 and Table 7. There was no correlation between HBc antigen and the concentration of the examined markers of bone turnover, inflammation and neutrophil trap.

## 4. Discussion

Due to reports in the literature on the frequent occurrence of low bone mass in both adults and children with congenital haemophilia [15,19,31,32,33], the authors analysed the concentrations of selected markers of bone turnover in the venous blood of adult patients with haemophilia A and B. They demonstrated a statistically significant difference in osteocalcin values compared to a healthy control group. In patients with congenital haemophilia, the median concentration of the described protein was 32.39 ng/mL, while in the control group it was 24.30 ng/mL. Osteocalcin is a sensitive marker of bone formation and elevated levels are found in patients with osteoporosis, among others [34]. The cut-off point obtained using the ROC plot was 26.41 ng/mL. Osteocalcin is a stimulant, which means that elevated osteocalcin levels are more common in patients with congenital haemophilia, and the results of the study confirm the increased risk of low bone mass in patients with haemophilia. Furthermore, the results of the analysis are consistent with those obtained at the paediatric department of Aristotle University of Thessaloniki (Greece), where 26 patients with haemophilia A (severe and moderate type) were studied [35].

Similar to us, the researchers from Greece did not detect a statistically significant difference between the values of osteocalcin concentrations in patients with severe and moderate forms of haemophilia. A limitation of the study from Aristotle University of Thessaloniki is that patients with mild forms of haemophilia were not included in the study group [35]. Higher osteocalcin levels in the blood of patients with congenital haemophilia were confirmed by Christofiridis and associates by studying 27 boys aged 4 to 17 years with haemophilia and comparing the concentration values obtained with an age- and sex-matched control group [36]. Moreover, the researchers pointed to a decrease in osteoprotegerin and an increase in the soluble receptor activator of nuclear factor κB ligand (sRANK-L) concentrations compared to the control group. The results suggest increased osteoclast activity with subsequent compensatory upregulation of the osteoblast function. Controversial results compared to those obtained in this study, in a study by Christofirdis and associates and in a study conducted at the University of Thessaloniki were obtained by researchers from Ankara (Turkey), where 44 paediatric patients with severe haemophilia A were studied [37]. The researchers indicated a reduction in osteocalcin levels in the study group compared to the control group. Despite the reduction in osteocalcin levels, increased bone remodelling in children with haemophilia was noted, and the degree of osteopenia indicated a statistically significant correlation with the degree of joint involvement. Results differing from ours were also obtained in the Haemophilia Comprehensive Care Centre of Agia Sofia Children’s Hospital in Athens, Greece, where the study group consisted of 51 boys aged 5 to 19 years [38].

The researchers assessed not only concentrations of bone turnover markers, but also performed dual energy X-ray absorptiometry (DXA) to evaluate bone mineral density in the lumbar spine and total body less head (TBLH). Mean values of total body bone mineral density without skull were lower in children with severe haemophilia compared to children with mild and moderate haemophilia, despite a reduction in osteocalcin levels in the blood of haemophilia patients. Another study contrasting with ours was conducted in the haemophilia centre of “Laikon” Hospital in Athens (Greece), where the prevalence of osteoporosis in adult patients with haemophilia was evaluated [39]. The study group consisted of 90 patients with a median age of 36 years. Inpatients with haemophilia, increased osteoclastic activity (increase in tartrate-resistant acid phosphatase isoform-5b-TRACP-5b, N-terminal cross-linking telopeptide of collagen type I-NTX, and C-terminal cross-linking telopeptide of collagen type I-CTX), but without increased bone formation (decrease in osteocalcin) were shown. In our study, there was no statistically significant difference in C-terminal telopeptide of collagen type I concentrations between the study and control groups. Interestingly, according to the researchers from the “Laikon” hospital in Athens, HIV infection was a predictor for the development of osteoporosis [39].

All the cited studies indicated changes in bone remodelling in patients with haemophilia compared to patients without haemorrhagic diathesis. However, the authors wish to emphasise that some of the cited studies were based on the analysis of bone turnover in developmental age, which could have influenced the values of concentrations of individual markers of bone turnover. Importantly, not every study reported increased osteoplastic activity associated with increased bone formation. In our study, osteocalcin was the only bone turnover marker in which there was a statistically significant difference in blood concentrations between the study and control groups. The differences in the concentrations of the other bone formation markers (bone alkaline phosphatase and N-terminal propeptide of type I collagen) and the bone resorption marker (C-terminal telopeptide of type I collagen) were not statistically significant. In our study, the levels of bone turnover markers were influenced by the prophylaxis used. In patients with haemophilia receiving prophylaxis with a deficient coagulation factor, the concentrations of C-terminal telopeptide of type I collagen and bone alkaline phosphatase reached lower values. On the other hand, blood osteocalcin concentrations were influenced by the applied anti-haemorrhagic therapy (lower concentrations occurred in patients treated on demand) and the form of haemophilia (in the mild form, osteocalcin concentrations were lower than in the case of severe haemophilia). On this basis, the authors conclude that blood concentrations of deficient coagulation factor (FVII/FIX) affect bone turnover and the risk of developing osteoporosis and osteopenia. The literature reports at least two pathways by which FVIII and FIX can affect the skeletal system: mitogenic osteoblast activity (mediated by thrombin) and osteoclast activity (mediated by cytokine) [40]. In addition, it is postulated that there is an indirect mechanism through the RANK-RANKL pathway and/or the Wnt/β-catenin pathway with the development of osteoporosis [41]. Osteoblasts and stem cells secrete the NF-kB receptor activator ligand (RANKL), which binds to the RANK receptor on the surface of osteoclasts and their precursors. This leads to the differentiation of the precursor cells into osteoclasts, as well as to their activation. Osteoblasts and their precursor cells secrete osteoprotegerin (OPG), which, by binding to RANKL, protects bone tissue from resorption. Therefore, the RANK/RANKL/OPG pathway influences bone metabolism in both physiological and pathological processes [42,43]. The possible influence of this mechanism on the development of osteoporosis in haemophilia is suggested by the studies of Chirstforidis et al. [36], which showed a decrease in the level of osteoprotegerin (OPG) and an increase in the soluble activator of the nuclear ligand receptor factor κB (sRANK-L) compared to the control group. The researchers, like us, obtained higher levels of osteocalcin in the study group. The studies of Christforidis et al. [36] suggest an increased activity of osteoclasts with subsequent compensatory regulation of osteoblast function, and confirm the pathogonomic mechanism that links rheumatoid arthritis and haemophilia [9,42,44,45].

There were no differences in the concentrations of the markers studied depending on the type of haemophilia (A and B). However, a tendency to higher blood levels of osteocalcin was observed in patients with haemophilia B, which may suggest the need for further research. It is important to note that, according to the literature, the concentration of bone turnover markers in patients with haemophilia may also be affected by viral status [12,19,31].

It has been shown that HCV mono-infection and co-infection with HIV and HCV in a patient with haemophilia affect the increase in bone alkaline phosphatase (BALP) levels [12]. The influence of viral infection on bone turnover markers was also shown in our study. It was observed that the previous infection with hepatitis C virus (presence of anti-HCV antibodies) decreases the value of C-terminal telopeptide of type I collagen and acid alkaline phosphatase concentrations, increases osteocalcin concentrations and correlates with ferritin concentration (*p* < 0.05). Exposure or vaccination against hepatitis B virus (the anti HBs antibodies) may have a stimulating effect on osteocalcin concentration and causes a decrease in vitamin D concentration. There was no statistically significant difference between the presence of anti-HBS total antibodies and markers of bone turnover. Due to the prevalence of vaccination against viral hepatitis (in Poland, since the 1980s, it has been a mandatory vaccination in the vaccination calendar), the studied groups are not equal, so the results of the comparison should be considered as a possible hypothesis that requires further verification.

The authors of this study, however, would like to point out that the centre where the research was conducted is the only centre in the Wielkopolska Province (Poland) that implements the “National Program of Treatment of Patients with Hemophilia and Related Hemorrhagic Diathesis” for adult patients. Nevertheless, due to the rarity of haemophilia (1 in 10,000 births [46], 1 in 12,300 inhabitants in Poland [47]) and the intensive development in the treatment of this bleeding disorder in recent years, it is not possible to gather an ideal group of patients, which we emphasize in relation to both our research and studies by other authors.

Analysing the available medical documentation of patients, the authors also noted the prevalence of vitamin D deficiency in patients with haemophilia A and B—sufficient vitamin D levels were noted in 1/5 of patients, which is in accordance with the results of other studies [15,19,31]. According to researchers, vitamin D deficiency may be dictated by geographical location and not only by disease entity—most studies report that vitamin D deficiency is common in the European population [48,49]. However, due to impaired bone metabolism and decreased bone mass among patients with haemophilia A and B, it is important to pay special attention to vitamin D supplementation in patients. Furthermore, due to the discrepancies in the concentrations of individual markers of bone turnover in patients with haemophilia, there is a need to continue studies assessing bone density and markers of bone turnover in this group of patients.

In our study, we attempted to find predictive markers for the development of haemophilic arthropathy. We observed a correlation between the occurrence of haemophilic arthropathy and an increase in the concentration of the inflammatory marker interleukin 6, an increase in the concentration of osteocalcin, and a decrease in the concentration of acid alkaline phosphatase (*p* < 0.05). The results also indicate a statistically significant difference in blood concentrations of citrullinated histone H3 (CH3), a marker of the so-called neutrophil extracellular trap (NET), between the study and control groups. The cut-off point for the stimulant CH3, obtained using the ROC plot, was 929.7 pg/mL. The neutrophil trap represents a mechanism through which neutrophils can fight pathogens, but at the same time, a study in Pittsburgh reported an association of neutrophil traps with the development of haemophilic arthropathy [30]. Recently, there have been other reports suggesting the influence of NETs on the pathogenesis of the development of haemophilic arthropathy, and experiments are underway to identify the immune pathways linking NETs with the development of haemophilic arthropathy [50].The plasma CH3 concentration is also affected by HCV infection and the applied anti-haemorrhagic therapy—in patients treated prophylactically with deficient coagulation factor, the concentration of this neutrophil extracellular trapping marker reaches lower values (*p* < 0.05). On this basis, the authors conclude that the concentration of citrullinated histone H3 may depend on the concentration of deficient coagulation factor in blood (FVIII/FIX). The authors of this study emphasize, however, that due to the relatively short history of recombinant coagulation factors, even those patients who currently take prophylactic factor deficiency did not have access to it in the early stages of life. The “Program for providing coagulation factors to patients with hemophilia and other bleeding disorders” in Poland was only established in 2001. In contrast, the ‘Prevention of Bleeding in Children with Haemophilia A and B’ program was not developed until 2008. It has been a drug program since 2012 and provides care to children with haemorrhagic diatheses [47]. For this reason, at our centre there are no adult patients treated with recombinant coagulation factor VIII/IX prophylactically throughout their lives. On this basis, it can be concluded that some of the complications in patients may have occurred before the initiation of treatment with recombinant coagulation factor. The authors of the study attempted to divide patients taking prophylactic factor deficiency into patients taking factor for a greater/lesser part of their lives. Unfortunately, such a division is not objective and would exclude older age groups from the study, which, according to the researchers, would adversely affect the reliability of the results. Since the development of complications depends, inter alia, on the duration of the disease, there is a need to include elderly patients in the study. At the same time, we would like to emphasize that in recent years there has been a marked improvement in the quality and life expectancy of patients with haemophilia. According to the literature, until 1960, the average life expectancy of a person with haemophilia was under 11 years of age. The introduction of clotting factor concentrates resulted in the extension of the life expectancy from 40 years (1960s) to 60–70 years [51,52]. Correct retrospective assessment of the effects of recombinant factor VIII and IX on the development of complications will only be possible if new generations have access to this factor throughout their lives. However, at this stage, a difference in the plasma concentrations of selected biomarkers can already be noticed in patients using factor VIII/IX prophylactically and “on demand”. The authors of the study speculate that these differences will be more significant in the future.

Recent reports have demonstrated a correlation between neutrophil extracellular traps and concentrations of D-dimer, tissue plasminogen activator (t-PA) and von Willenbrand factor (vW) [53]. For this reason, in the authors’ opinion, there is a need to continue studies on the influence of neutrophil traps and inflammatory markers on the development of complications (including haemophilic arthropathy and reduced bone density) in haemophilia.

## 5. Conclusions

Our study shows that the concentration of citrated histone CH3 in the plasma, a marker of extracellular neutrophilic trap, is higher in patients with congenital haemophilia compared to patients without a congenital bleeding disorder. Moreover, the concentration of the marker becomes higher in patients not taking prophylactic deficiency of coagulation factor. Elevation of serum osteocalcin, a marker of bone tissue formation, is more common in haemophilia patients than in healthy patients. The concentration of bone turnover markers in plasma may depend on the applied anti-haemorrhagic prophylaxis, the severity of the underlying disease, and on the development of haemophilic arthropathy.

## Figures and Tables

**Table 1:** Characteristics of the study group including the type and severity of haemophilia and the type of management.

		*n*	%
Type of Haemophilia	A	48	80.0
	B	12	20.0
Routine Management	On-demand therapy	27	45.0
	Secondary prophylactic therapy	33	55.0
Severity of Haemophilia	Severe	42	70.0
	Moderate	9	15.0
	Mild	9	15.0

**Table 2:** Comparison of concentrations of interleukin 6 (IL-6); citrullinated histone CH3 (marker of neutrophil extracellular trap); markers of bone formation (osteocalcin (BGLAP), bone alkaline phosphatase (BALP), N-terminal propeptide of type I procollagen (P1NP)); and the bone resorption marker C-terminal telopeptide of type I collagen (C1CP) between the study group and the control group (median and Q1–Q3).

	Study Group *n* = 60	Control Group *n* = 30	*p*-Value
	M [Q1–Q3]	M [Q1–Q3]	
IL-6 [pg/mL]	214.1 [158.4–322.8]	216.5 [163.4–267.3]	0.716
CH3 [pg/mL]	1019.2 [909.8–1130.1]	859.3 [798.0–934.3]	<0.001 *
CICP [ng/mL]	173.1 [129.4–210.8]	165.8 [145.9–176.6]	0.250
PINP [ng/mL]	1057.7 [905.9–1198.7]	1121.4 [992.6–1256.7]	0.154
BALP [U/L]	292.8 [261.8–350.9]	328.1 [263.7–354.0]	0.138
BGLAP [ng/mL]	32.4 [26.6–37.6]	24.3 [21.5–29.6]	<0.001 *

* *p*-value < 0.05 for the Mann–Whitney test.

**Table 3:** Comparison of concentrations of interleukin 6 (IL-6); citrullinated histone CH3 (marker of neutrophil extracellular trap); markers of bone formation (osteocalcin (BGLAP), bone alkaline phosphatase (BALP), N-terminal propeptide of type I procollagen (P1NP)); bone resorption marker C-terminal telopeptide of type I collagen (C1CP); and vitamin D, ferritin and serum calcium, depending on the haemorrhagic prophylaxis used (on-demand/prophylactic therapy) (median and Q1–Q3).

	On-Demand Therapy *n* = 27	Prophylactic Therapy *n* = 33	*p*-Value
	M [Q1–Q3]	M [Q1–Q3]	
IL-6 [pg/mL]	181.4 [155.0–354.6]	244.8 [169.3–316.8]	0.305
CH3 [pg/mL]	1114.4 [934.3–1149.8]	952.5 [875.6–1057.4]	0.008 *
CICP [ng/mL]	191.2 [160.2–224.3]	161.6 [126.4–189.6]	0.015 *
PINP [ng/mL]	1117.6 [882.9–1214.6]	1030.9 [927.5–1178.9]	0.888
BALP [U/L]	322.5 [281.3–356.4]	280.9 [254.2–338.0]	0.038 *
BGLAP [ng/mL]	28.0 [24.5–32.9]	35.9 [28.9–39.9]	0.017 *
Vitamin D [ng/mL]	21.1 [16.3–24.7]	17.5 [14.1–25.2]	0.142
Calcium [mmol/L]	2.5 [2.5–2.6]	2.5 [2.4–2.6]	0.175
Ferritin [ng/mL]	93.4 [42.0–175.7]	93.0 [65.2–236.9]	0.357

* *p*-value < 0.05 for the Mann–Whitney test.

**Table 4:** Comparison of concentrations of interleukin 6 (IL-6); citrullinated histone CH3 (marker of neutrophil extracellular trap); markers of bone formation (osteocalcin (BGLAP), bone alkaline phosphatase (BALP), N-terminal propeptide of type I procollagen (P1NP)); and the bone resorption marker C-terminal telopeptide of type I collagen (C1CP), according to the severity of haemophilia (mild/moderate, severe) (median and Q1–Q3).

	Mild and Moderate *n* = 18	Severe *n* = 42	*p*-Value
	M [Q1–Q3]	M [Q1–Q3]	
IL-6 [pg/mL]	164.6 [155.0–228.3]	247.0 [169.3–328.7]	0.131
CH3 [pg/mL]	1048.9 [959.4–1145.2]	971.4 [875.6–1101.3]	0.118
CICP [ng/mL]	188.3 [163.1–224.3]	166.2 [126.4–208.8]	0.070
PINP [ng/mL]	1113.8 [889.2–1194.2]	1015.6 [912.2–1212.0]	0.955
BALP [U/L]	311.0 [281.3–348.1]	288.7 [250.7–352.7]	0.208
BGLAP [ng/mL]	27.1 [24.5–36.3]	32.7 [27.9–38.5]	0.183
Vitamin D [ng/mL]	20.7 [17.9–24.7]	19.0 [14.6–24.6]	0.348
Calcium [mmol/L]	2.5 [2.5–2.6]	2.5 [2.4–2.6]	0.354
Ferritin [ng/mL]	65.0 [42.0–115.9]	98.7 [68.5–228.8]	0.055

**Table 5:** Comparison of concentrations of interleukin 6 (IL-6); citrullinated histone CH3 (marker of neutrophil extracellular trap); markers of bone formation (osteocalcin (BGLAP), bone alkaline phosphatase (BALP), N-terminal propeptide of type I procollagen (P1NP)); bone resorption marker-C-terminal telopeptide of type I collagen (C1CP); and vitamin D, ferritin and serum calcium between patients with and without haemophilic arthropathy (median and Q1–Q3).

	With Arthropathy *n* = 42	Without Arthropathy *n* = 18	*p*-Value
	M [Q1–Q3]	M [Q1–Q3]	
IL-6 [pg/mL]	254.1 [169.3–362.5]	164.6 [135.8–211.4]	0.020 *
CH3 [pg/mL]	957.4 [880.7–1101.3]	1062.6 [987.3–1146.3]	0.108
CICP [ng/mL]	167.8 [127.1–212.1]	182.6 [163.1–207.3]	0.397
PINP [ng/mL]	1015.6 [908.4–1178.9]	1157.2 [882.9–1214.6]	0.429
BALP [U/L]	284.0 [254.2–338.0]	332.3 [291.0–353.9]	0.028 *
Osteocalcin [ng/mL]	34.9 [28.0–39.9]	26.7 [23.3–30.3]	0.002 *
Vitamin D [ng/mL]	19.1 [15.0–25.8]	21.1 [17.0–23.5]	0.525
Calcium [mmol/L]	2.5 [2.4–2.6]	2.5 [2.5–2.6]	0.388
Ferritin [ng/mL]	99.6 [0.23–0.63]	66.4 [44.9–112.4]	0.148

* *p*-value < 0.05 for the Mann–Whitney test.

**Table 6:** Comparison of concentrations of interleukin 6 (IL-6); citrullinated histone CH3 (marker of neutrophil extracellular trap); markers of bone formation (osteocalcin (OT), bone alkaline phosphatase (BALP), N-terminal propeptide of type I procollagen (P1NP)); bone resorption marker-C-terminal telopeptide of type I collagen (C1CP); and vitamin D, ferritin and serum calcium, depending on the presence of serum anti-HBS antibodies (reactive or non-reactive result) (median and Q1–Q3).

	antyHBs Positive *n* = 48	antyHBs Negative *n* = 6	*p*-Value
	M [Q1–Q3]	M [Q1–Q3]	
IL-6 [pg/mL]	247.0 [168.4–358.1]	149.4 [132.0–211.1]	0.107
CH3 [pg/mL]	997.0 [908.7–1131.5]	1013.0 [910.3–1114.4]	0.869
CICP [ng/mL]	169.5 [129.4–210.5]	169.3 [122.0–189.9]	0.670
PINP [ng/mL]	1083.2 [913.5–1213.3]	916.1 [882.9–1057.7]	0.137
BALP [U/L]	298.0 [258.5–351.8]	290.2 [289.1–303.4]	0.527
BGLAP [ng/mL]	32.7 [27.9–38.7]	24.3 [23.3–27.3]	0.006 *
Vitamin D [ng/mL]	18.5 [14.8–23.5]	37.9 [20.3–40.1]	0.011 *
Calcium [mmol/L]	2.5 [2.4–2.6]	2.5 [2.5–2.6]	0.793
Ferritin [ng/mL]	94.2 [64.0–224.6]	133.6 [63.5–175.7]	0.988

* *p*-value < 0.05 for the Mann–Whitney test.

**Table 7:** Comparison of concentrations of interleukin 6 (IL-6); citrullinated histone CH3 (marker of neutrophil extracellular trap); markers of bone formation (osteocalcin (OT), bone alkaline phosphatase (BALP), N-terminal propeptide of type I procollagen (P1NP)); bone resorption marker-C-terminal telopeptide of type I collagen (C1CP); and vitamin D, ferritin and serum calcium, depending on the presence of anti-HCV antibodies in the serum (reactive or non-reactive result) (median and Q1–Q3).

	antyHCV Positive n = 28	antyHCV Negative n = 29	*p*-Value
	M [Q1–Q3]	M [Q1–Q3]	
IL-6 [pg/mL]	228.3 [175.4–345.2]	189.5 [155.0–260.7]	0.367
CH3 [pg/mL]	952.0 [860.5–1076.0]	1054.6 [939.4–1146.3]	0.034 *
CICP [ng/mL]	131.6 [119.8–199.1]	191.2 [161.6–220.0]	0.020 *
PINP [ng/mL]	974.8 [886.1–1175.1]	1090.8 [927.5–1212.0]	0.271
BALP [U/L]	281.1 [250.2–330.5]	322.5 [288.1–353.0]	0.046 *
Osteocalcin [ng/mL]	34.9 [32.0–38.2]	28.0 [25.3–32.9]	0.022 *
Vitamin D [ng/mL]	19.2 [15.7–31.4]	19.4 [15.3–23.2]	0.640
Calcium [mmol/L]	2.5 [2.5–2.6]	2.5 [2.5–2.6]	0.581
Ferritin [ng/mL]	119.7 [88.0–254.7]	66.1 [44.1–103.7]	0.003 *

* *p*-value < 0.05 for the Mann–Whitney test.

## Data Availability

Due to the nature of this research, participants of this study did not agree for their data to be shared publicly, so supporting data are not available.
